# Antibacterial and Cytotoxic Study of Hybrid Films Based on Polypropylene and NiO or NiFe_2_O_4_ Nanoparticles

**DOI:** 10.3390/ijms242317052

**Published:** 2023-12-02

**Authors:** Karen L. Rincon-Granados, América R. Vázquez-Olmos, Adriana-Patricia Rodríguez-Hernández, Gina Prado-Prone, Margarita Rivera, Vicente Garibay-Febles, Yara C. Almanza-Arjona, Roberto Y. Sato-Berrú, Esther Mata-Zamora, Phaedra S. Silva-Bermúdez, Alejandro Vega-Jiménez

**Affiliations:** 1 Instituto de Ciencias Aplicadas y Tecnología, Universidad Nacional Autónoma de México, Circuito Exterior s/n, Ciudad de México 04510, Mexico; karenloraine08@gmail.com (K.L.R.-G.); roberto.sato@icat.unam.mx (R.Y.S.-B.); esther.mata@icat.unam.mx (E.M.-Z.); 2 Laboratorio de Genética Molecular, División de Estudios de Posgrado e Investigación de la Facultad de Odontología, Universidad Nacional Autónoma de México, Ciudad de México 04510, Mexico; aprh_gm@fo.odonto.unam.mx; 3 Facultad de Odontología, División de Estudios de Posgrado e Investigación, Universidad Nacional Autónoma de México, Circuito Exterior s/n, Ciudad Universitaria, Ciudad de México 04510, Mexico; gpradoprone@comunidad.unam.mx (G.P.-P.); dr.vegalex@gmail.com (A.V.-J.); 4 Instituto de Física, Universidad Nacional Autónoma de México, Ciudad de México 04510, Mexico; mrivera@fisica.unam.mx; 5 Instituto Mexicano del Petróleo, Eje Central Lázaro Cárdenas Norte 152 Col. San Bartolo Atepehuacan, Ciudad de México 07730, Mexico; vgaribay@imp.mx; 6 Tecnologico de Monterrey, Escuela de Ingeniería y Ciencias, Monterrey 64849, Nuevo León, Mexico; yara.almanza@tec.mx; 7 Unidad de Ingeniería de Tejidos, Terapia Celular y Medicina Regenerativa, Instituto Nacional de Rehabilitación Luis Guillermo Ibarra Ibarra, Ciudad de México 14389, Mexico; phaedrasilva@yahoo.com

**Keywords:** hybrid, films, nanoparticles, polypropylene, antibacterial, cytotoxicity

## Abstract

This study presents an in vitro analysis of the bactericidal and cytotoxic properties of hybrid films containing nickel oxide (NiO) and nickel ferrite (NiFe_2_O_4_) nanoparticles embedded in polypropylene (PP). The solvent casting method was used to synthesize films of PP, PP@NiO, and PP@NiFe_2_O_4_, which were characterized by different spectroscopic and microscopic techniques. The X-ray diffraction (XRD) patterns confirmed that the small crystallite sizes of NiO and NiFe_2_O_4_ NPs were maintained even after they were incorporated into the PP matrix. From the Raman scattering spectroscopy data, it was evident that there was a significant interaction between the NPs and the PP matrix. Additionally, the Scanning Electron Microscopy (SEM) analysis revealed a homogeneous dispersion of NiO and NiFe_2_O_4_ NPs throughout the PP matrix. The incorporation of the NPs was observed to alter the surface roughness of the films; this behavior was studied by atomic force microscopy (AFM). The antibacterial properties of all films were evaluated against *Pseudomonas aeruginosa* (ATCC^®^: 43636™) and *Staphylococcus aureus* (ATCC^®^: 23235™), two opportunistic and nosocomial pathogens. The PP@NiO and PP@ NiFe_2_O_4_ films showed over 90% bacterial growth inhibition for both strains. Additionally, the effects of the films on human skin cells, such as epidermal keratinocytes and dermal fibroblasts, were evaluated for cytotoxicity. The PP, PP@NiO, and PP@NiFe_2_O_4_ films were nontoxic to human keratinocytes. Furthermore, compared to the PP film, improved biocompatibility of the PP@NiFe_2_O_4_ film with human fibroblasts was observed. The methodology utilized in this study allows for the production of hybrid films that can inhibit the growth of Gram-positive bacteria, such as *S. aureus*, and Gram-negative bacteria, such as *P. aeruginosa*. These films have potential as coating materials to prevent bacterial proliferation on surfaces.

## 1. Introduction

In recent decades, there has been a growing global concern regarding the rise in antibiotic-resistant bacterial strains, affecting both Gram-positive and Gram-negative bacteria [1]. *Staphylococcus aureus* and *Pseudomonas aeruginosa* are highly resistant strains involved in nosocomial diseases, which includes strains such as methicillin-resistant *Staphylococcus aureus* (MRSA) [2]. *S. aureus* is strongly associated with infections, such as enteric infections, toxic shock syndrome, nosocomial pneumonia, and bacteremia, among others. Patients infected with MRSA may experience a delayed recovery or even the risk of death [3]. In contrast, *P. aeruginosa* is responsible for causing hospital-acquired infections that can have severe implications for immunocompromised patients who have undergone resuscitation, received anesthesia, or had respiratory or hemodynamic issues [4]. Patients requiring mechanical ventilation, particularly during the pandemic, are also at risk [5]. This bacterium is highly resistant to antibiotics and disinfectants, which can increase mortality rates in patients suffering from pneumonia or chronic lung infections [6]. Consequently, it is crucial to discover new ways to treat and prevent bacterial infections; in this sense, nanotechnology has contributed to developing new antimicrobial agents [7,8], focusing on the use of different nanomaterials that, due to their physical and chemical properties, inhibit bacterial growth through different action mechanisms [9]. In these nanomaterials, nanostructured metal oxides have gained significant interest due to the growth inhibition of these nanostructures against different bacteria (evaluated in vitro and provided by international culture collections), such as nickel oxide (NiO), which exhibits antibacterial activity against *Escherichia coli*, *Bacillus subtilis*, *S. aureus*, and *P. aeruginosa* [10,11]. On the other hand, the study of mixed metal oxides has also generated interest, as is the case of nickel ferrite (NiFe_2_O_4_), which has shown antibacterial activity against *Streptococcus pyogenes*, *Salmonella typhimurium*, *E. coli S. aureus*, and *P. aeruginosa* [11,12]. However, the use of these nanoparticles is limited due to their nature since they are commonly obtained as powders or colloidal solutions [13,14]; therefore, using them as coating materials to inhibit the spread of bacteria is not applicable. A promising solution for applying nanoparticles is to create hybrid materials, which are formed by the synergistic combination of organic and inorganic components at the molecular level or nanometric scale [15]. The use of hybrid materials, particularly hybrid films, as antibacterial agents has not been thoroughly researched. However, studies have been conducted on the antibacterial properties of hybrid materials made from natural polymers such as nanocellulose, and metal oxide NPs such as ZnO, TiO_2_, CuO, MgO, and Fe_3_O_4_. These studies have shown that these materials have a bactericidal effect against Gram-positive and Gram-negative strains [16]. Moreover, poly (methyl methacrylate) (PMMA) nanocomposites containing CuO and ZnO NPs have also been researched and proven effective against *S. aureus* (V329) and *E. coli* (JM101) [17]. These findings are significant as they open up opportunities for creating hybrid materials for biomedical applications that can effectively combat infections caused by microorganisms. In this sense, it is proposed to obtain a film-type hybrid material between a polymer matrix and nanoparticles (polymer@NPs) that allows the antibacterial properties to be extended and applied to surfaces [18].

Therefore, as a polymer matrix of a hybrid material, polypropylene (PP) arouses interest since it is a polymer widely used in the healthcare industry due to its resistance to chemicals, heat, and radiation, as well as its transparency, gas and liquid barrier properties, impact resistance, flexibility, and cost-effectiveness. PP-based products are prevalent in medical devices, packaging, and solid and liquid pharmaceutical delivery systems [19,20]. Modifications can render these polymers suitable not only for their physicochemical and mechanical properties but also as antibacterial materials, potentially addressing the growing demand for preventing hospital-acquired bacterial infections.

This study explores the antibacterial properties of hybrid films made from polypropylene (PP), nickel oxide (NiO), and nickel ferrite (NiFe_2_O_4_) nanoparticles. The films are tested in vitro against *S. aureus* and *P. aeruginosa*. Additionally, the research evaluates the cytotoxicity of these synthesized films against human keratinocytes and dermal fibroblasts.

## 2. Results

The properties of the obtained films (PP, PP@NiO, and PP@NiFe_2_O_4_) were studied by several characterization techniques, including X-ray diffraction (XRD), Raman spectroscopy, scanning electron microscopy (SEM), energy-dispersive X-ray spectroscopy (EDS), atomic force microscopy (AFM), and contact angle measurements. The obtained results are presented below.

### 2.1. X-ray Diffraction Patterns of Films

**Figure 1:**
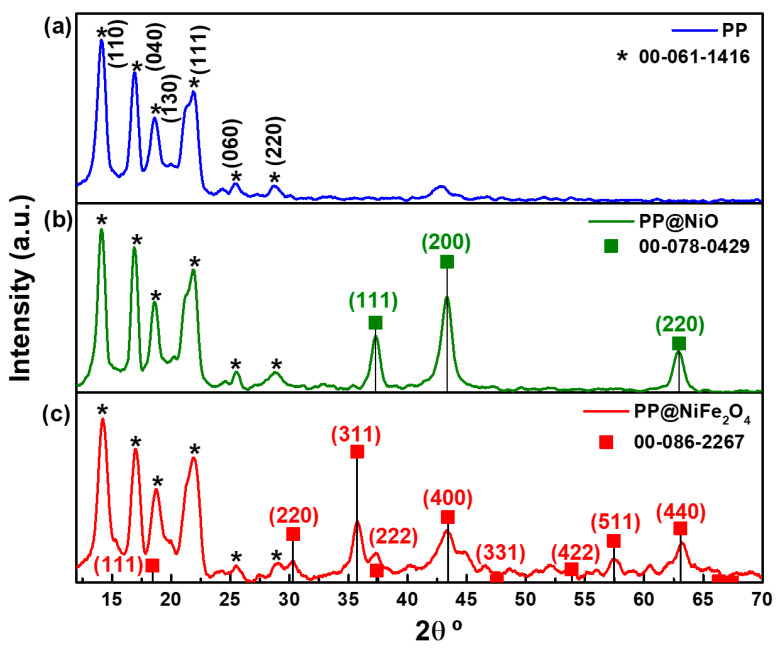
X-ray diffraction patterns of films: (**a**) PP, (**b**) PP@NiO, and (**c**) PP@NiFe_2_O_4_. ICDD cards for PP, NiO and NiFe_2_O_4_ are included.

The X-ray diffraction (XRD) patterns of the PP@NiO, PP@NiFe_2_O_4_ hybrid films, and the PP film are shown in Figure 1. The samples exhibited the characteristic diffraction peaks of PP (ICDD 00-061-card) [21]. Furthermore, the XRD patterns of the PP@NiO and PP@NiFe_2_O_4_ hybrid films revealed the presence of the diffraction peaks characteristic of NiO (ICDD 00-078-0429) and NiFe_2_O_4_ (ICDD 00-086-2267) [11], respectively. This indicates that the NPs have been effectively incorporated into the polymeric matrix.

The average crystallite size of each metallic oxide NPs in the hybrid films was determined using the Scherrer’s equation. It was found that the PP@NiO film had an average crystallite size of 7 ± 0.4 nm and the PP@NiFe_2_O_4_ film had an average crystallite size of 6 ± 1.1 nm. The sizes of the nanoparticles in the hybrid film were similar to those observed in the XRD patterns of the individual oxides (7 ± 0.8 nm for NiO and 5 ± 0.9 nm for NiFe_2_O_4_) [11]. The experimental processing did not impact the size of the nanocrystallite.

### 2.2. Raman Characterization of Films

**Figure 2:**
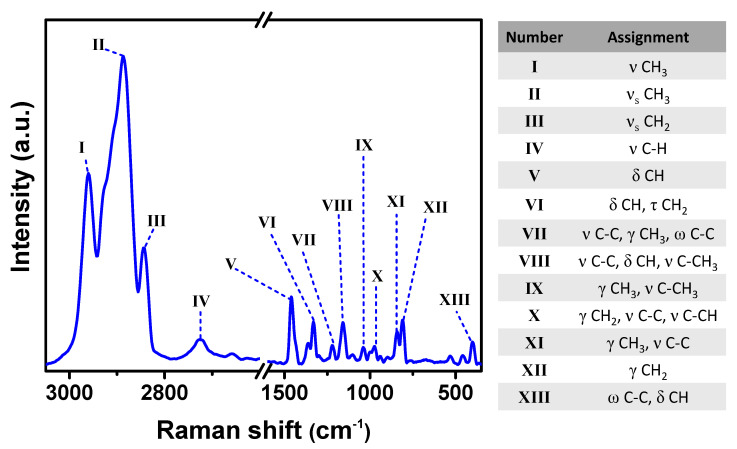
Raman spectrum of PP film and the assignment of signals to the respective vibration.

The Raman spectrum of PP film in Figure 2 displays the vibrational modes assigned to the polymer’s functional groups, as reported in the literature [22,23].

**Figure 3:**
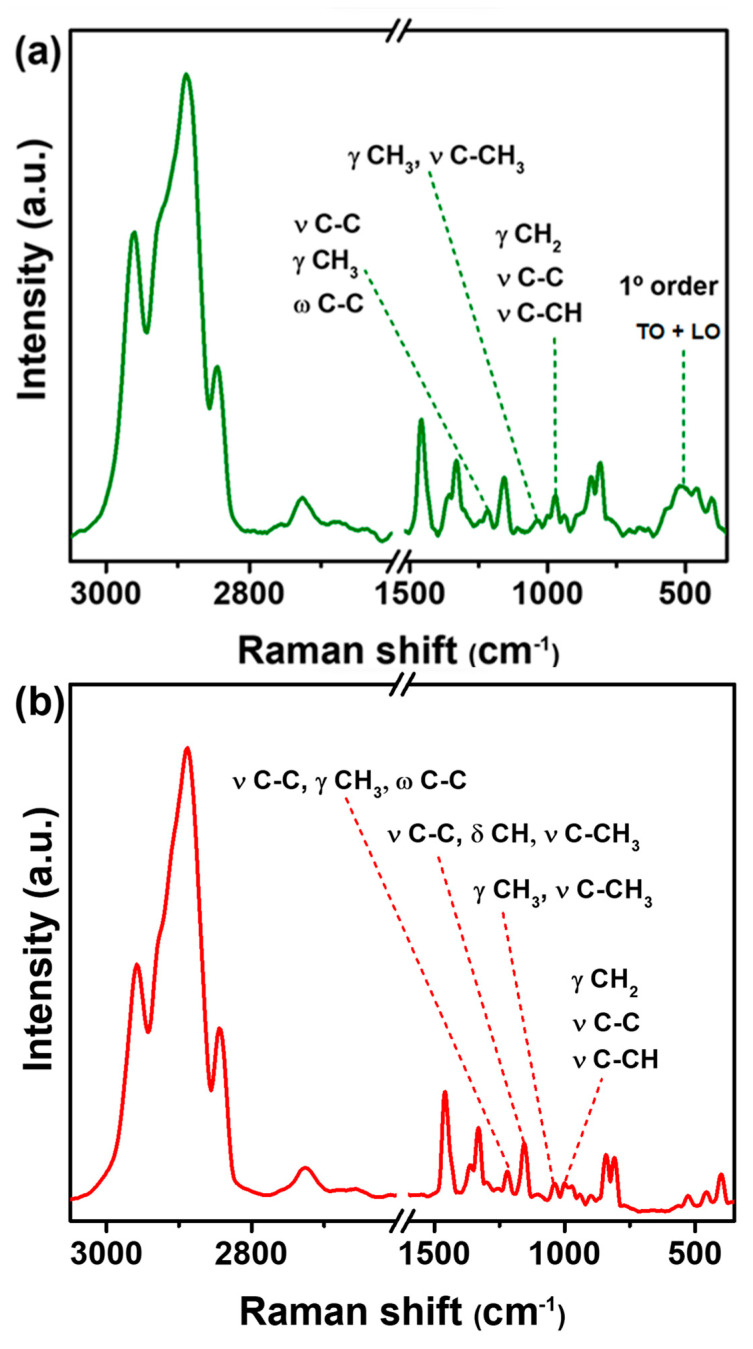
Raman spectra of hybrid films of (**a**) PP@NiO and (**b**) PP@NiFe_2_O_4_.

The thermal treatment that PP underwent when it became a film did not impact the vibrational modes of its functional groups. Figure 3 shows the Raman spectra of PP@NiO and PP@NiFe_2_O_4_ hybrid films. The PP@NiO hybrid film (Figure 3a) exhibited the main bands corresponding to the vibrations of the PP polymer and the NiO NPs. When comparing this hybrid film to the PP film (shown in Figure 2), changes can be observed in certain bands of the functional groups of PP, such as the band at 1043 cm^−1^ assigned to γ CH_3_, ν C -CH_3_, which is found at very low intensity in the PP@NiO hybrid film. The bands at 1357 cm^−1^ (ν CH_3_, σ CH), 1215 (ν C-C, γ CH_3_, ꞷ C-C), and 983 cm^−1^ (γ CH_3_, ν C-C) presented displacements at 1364, 1208, and 973 cm^−1^ in the hybrid film, indicating that the interaction with the NPs is associated with the CH_3_, CH groups, and the carbon chain of PP. The peaks at 420 and 500 cm^−1^ are assigned to first-order transverse optical (TO) and longitudinal optical (LO) phonon modes of NiO, respectively, according to the literature [11,24,25]. The signal with a maximum at 511 cm^−1^ is the envelope of (TO + LO) phonon modes of the NiO. The observed shift suggests a strong interaction between the NPs and the PP polymeric matrix. The comparison of the Raman spectra of the isolated NiO NPs and the PP@NiO hybrid film is presented in the supporting information in Figure S1. On the other hand, as depicted in Figure 3b, in the Raman spectrum of PP@NiFe_2_O_4_ hybrid film, the characteristic signals of the NiFe_2_O_4_ NPs are not observed. It could be thought that the NPs were not embedded in the polymer matrix; however, the results shown in the XRD (Figure 1) indicate the effective formation of the hybrid film between the NPs and the polymer. Therefore, it is suggested that the Raman technique for this hybrid film was not the most suitable for observing NPs. Despite this, displacements are observed and compared to the PP film. These bands include 1215 cm^−1^ (ν C-C, γ CH_3_, ꞷ C-C), 1155 cm^−1^ (ν C-C, δ CH, ν C-CH_3_), 1043 cm^−1^ (γ CH_3_, ν C-CH_3_), and 983 cm^−1^ (γ CH_3_, ν C-C) at 1221, 1150, 1035, and 992 cm^−1^, respectively. This indicates that the NiFe_2_O_4_ NPs also interact with the CH_3_, CH groups, and the carbon chain of PP in the hybrid film. To visualize the NiFe_2_O_4_ NPs in the PP@ NiFe_2_O_4_ hybrid film, a Raman spectrum using a higher laser power (3 mW) was obtained. This helped to detect a signal at 696 cm^−1^ associated with the symmetric stretching of the Fe-O tetrahedral bonds (A_1g_) of the NiFe_2_O_4_ NPs. However, the use of higher laser power caused degradation of the polymeric matrix, which led to a loss of definition of the representative bands of the PP. For a better understanding, refer to Figure S2 in the supporting information, which presents the Raman spectra of NiFe_2_O_4_ NPs compared to PP@NiFe_2_O_4_ hybrid films at two different laser powers.

### 2.3. Scanning Electron Microscopy of Films

**Figure 4:**
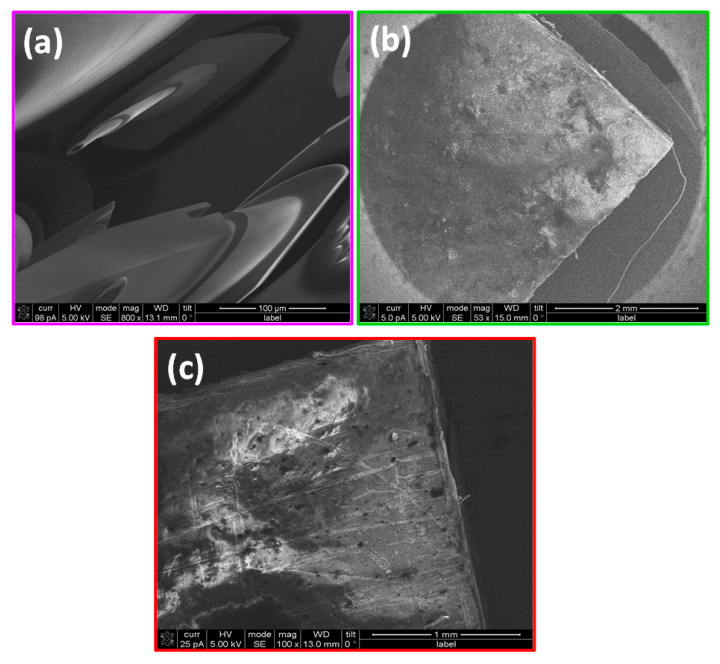
SEM images of the films of (**a**) PP, (**b**) PP@NiO, and (**c**) PP@NiFe_2_O_4_.

In Figure 4, SEM images of the films of PP, PP@NiO, and PP@NiFe_2_O_4_ are shown. The surface of the PP film in Figure 4a looks degraded due to the voltage of 30 kV, which was used to obtain the image. Upon comparing the PP film with the hybrid films illustrated in Figure 4b,c, it is evident that the surface of these films is rougher. Additionally, the hybrid films do not show any signs of degradation, even when exposed to the same voltage of 30 kV. This observation suggests that the metallic oxide NPs that are incorporated in the PP matrix not only cause roughness but also provide stability to the polymeric films.

**Figure 5:**
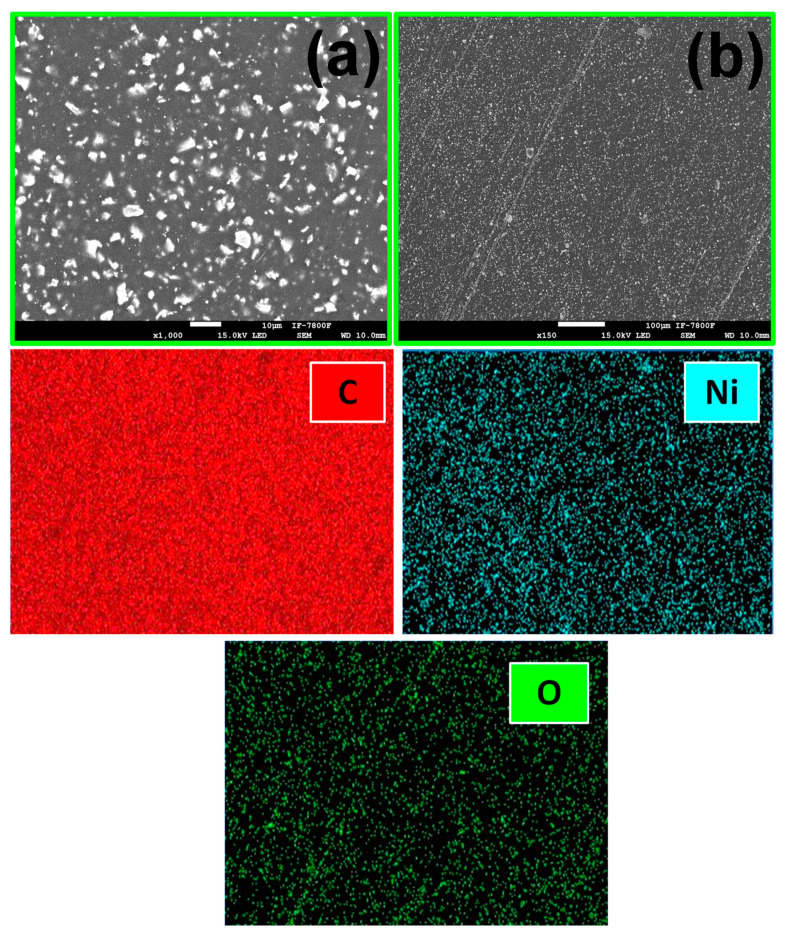
SEM images of PP@NiO at different magnifications: (**a**) 1000× and (**b**) 150×. The bottom three images show the chemical mapping of the film surface for C, Ni, and O.

**Figure 6:**
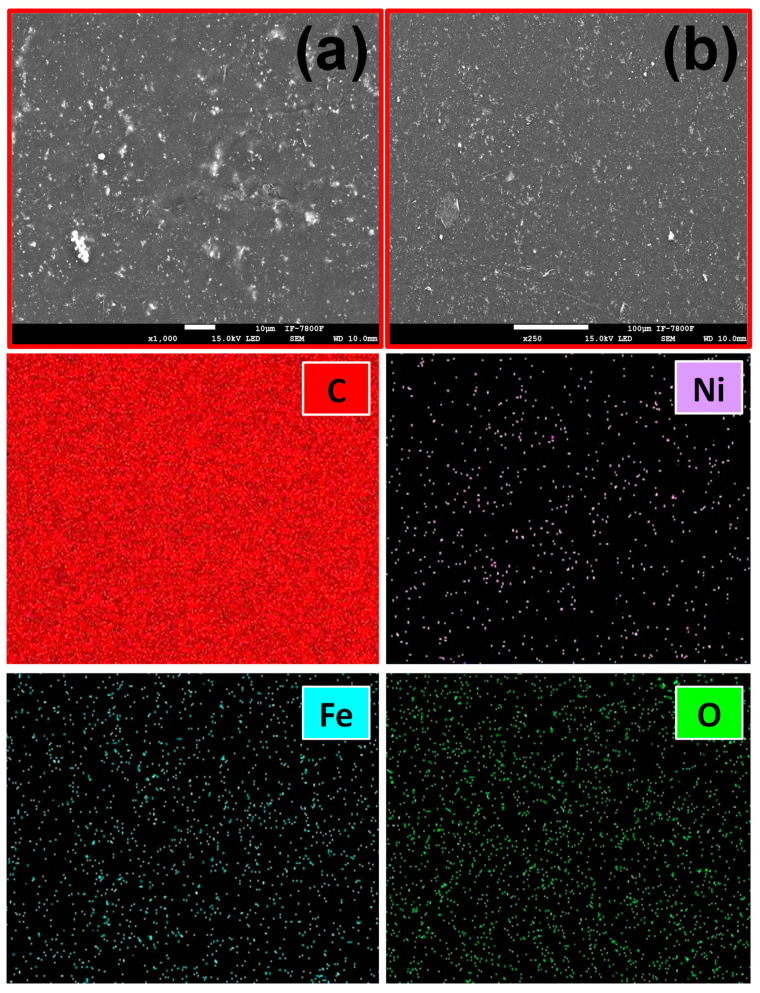
SEM images of PP@NiFe_2_O_4_ at different magnifications: (**a**) 1000× and (**b**) 150×. The bottom four images show the chemical mapping of the film surface for C, Ni, Fe, and O.

In addition, chemical mapping of the surface of the films is shown in Figure 5 and Figure 6 for PP@NiO and PP@NiFe_2_O_4_, where it is evident that the NPs are homogeneously distributed in the PP surface.

**Figure 7:**
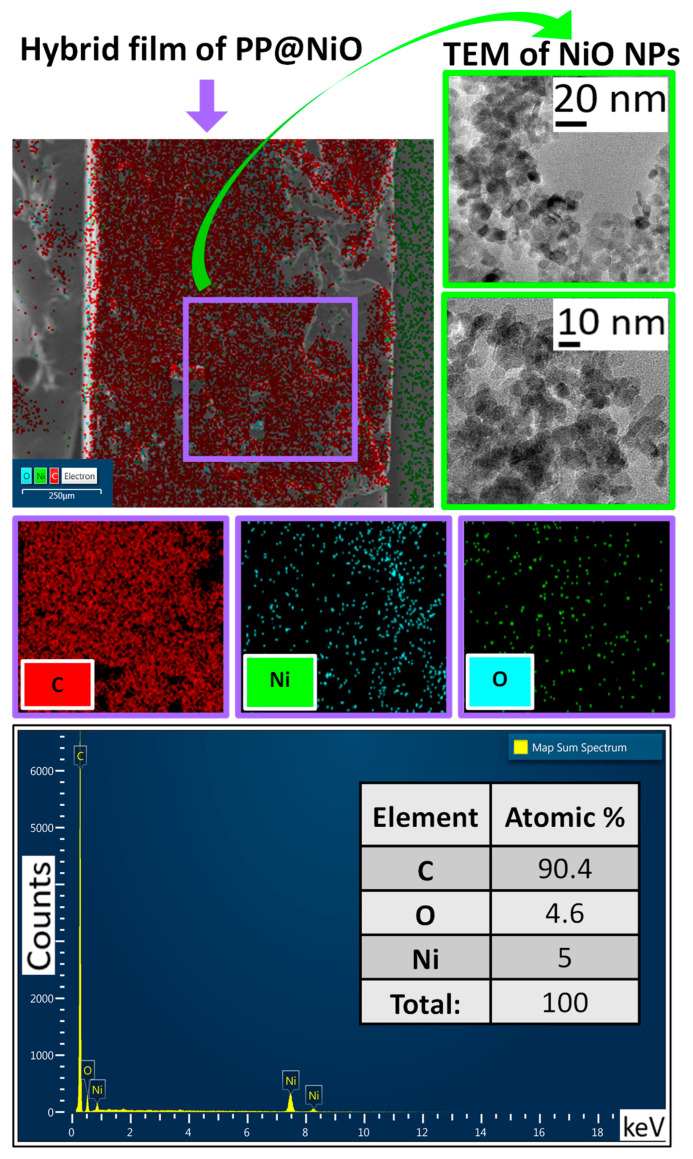
Chemical mapping of the PP@NiO hybrid film (cross-section), with chemical elements represented in diverse colors along with elemental quantification. Additionally, TEM images of the NiO NPs incorporated into the polymer are included. This provides a visual representation of the distribution and composition of the elements in the hybrid film.

**Figure 8:**
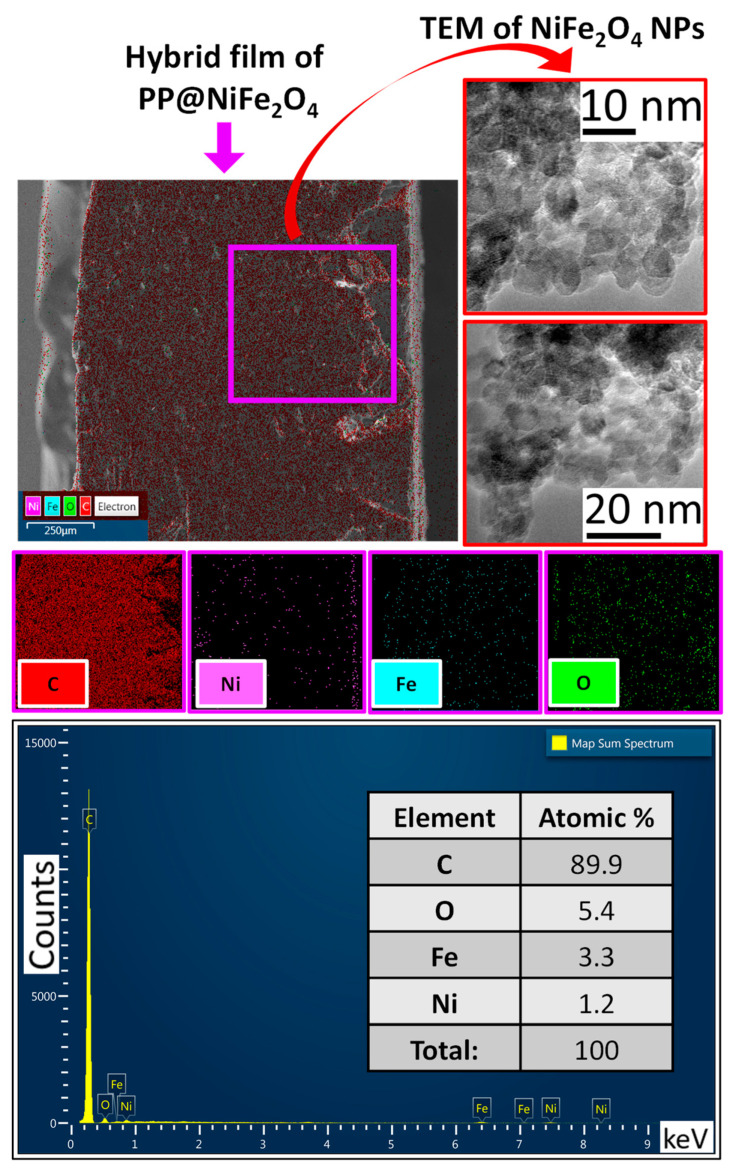
Chemical mapping of the PP@NiFe_2_O_4_ hybrid film (cross-section), with chemical elements represented in diverse colors along with elemental quantification. Additionally, TEM images of the NiFe_2_O_4_ NPs incorporated into the polymer are included. This provides a visual representation of the distribution and composition of the elements in the hybrid film.

Additionally, SEM microscopy was used to conduct a cross-sectional analysis of the hybrid films of PP@NiO and PP@NiFe_2_O_4_. Chemical mapping and quantification of chemical elements were employed to observe the distribution of the nanoparticles (NPs) in each film. Figure 7 and Figure 8 present the chemical mappings of the PP@NiO and PP@NiFe_2_O_4_ hybrid films; furthermore, TEM images of the NPs previously synthesized and incorporated into the polymer are presented. The NPs size distribution was reported previously [11]. The chemical mapping of the cross-sections of the hybrid films (PP@NiO and PP@NiFe_2_O_4_) reveals a uniform distribution of NPs throughout the polymer, particularly on the film surfaces, as confirmed in Figure 5 and Figure 6. This uniform distribution is favorable for interactions with bacterial strains. Additionally, the nickel quantification (Ni) in the PP@NiO hybrid film corresponded to the weight percentage of NPs added, which is approximately 10% (Ni = 5.0%, O = 4.6%). Similarly, the Ni and Fe quantification in the PP@NiFe_2_O_4_ hybrid film is also in agreement with the amount of NPs incorporated into the respective hybrid film, which is around 10% (Ni = 1.2%, Fe = 3.3%, O = 5.4%).

**Figure 9:**
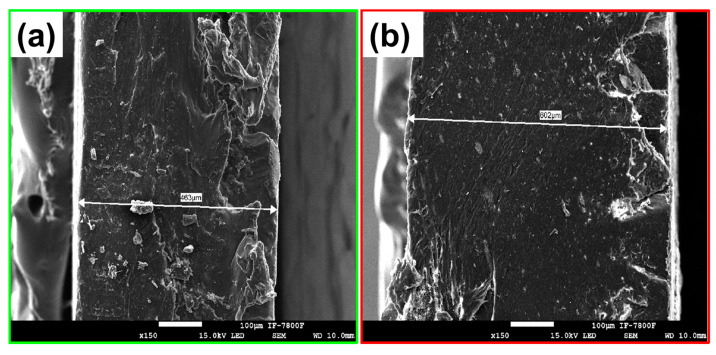
Cross-section images of (**a**) PP@NiO and (**b**) PP@NiFe_2_O_4_ hybrid films, obtained by SEM.

Additionally, a cross-section of the hybrid films was carried out and analyzed by SEM (Figure 9). The thickness of the hybrid films was 463 μm and 602 μm for PP@NiO and PP@NiFe_2_O_4_, respectively. Furthermore, a micro vernier was used to corroborate the thickness of the films. The thickness was 508 ± 0.7 μm and 651 ± 0.8 μm for PP@NiO and PP@NiFe_2_O_4_, respectively. These measurements are the average of three different films for each system. All films exhibit enough uniformity in their thickness, leading to good reproducibility.

### 2.4. Atomic Force Microscopy of Films

**Figure 10:**
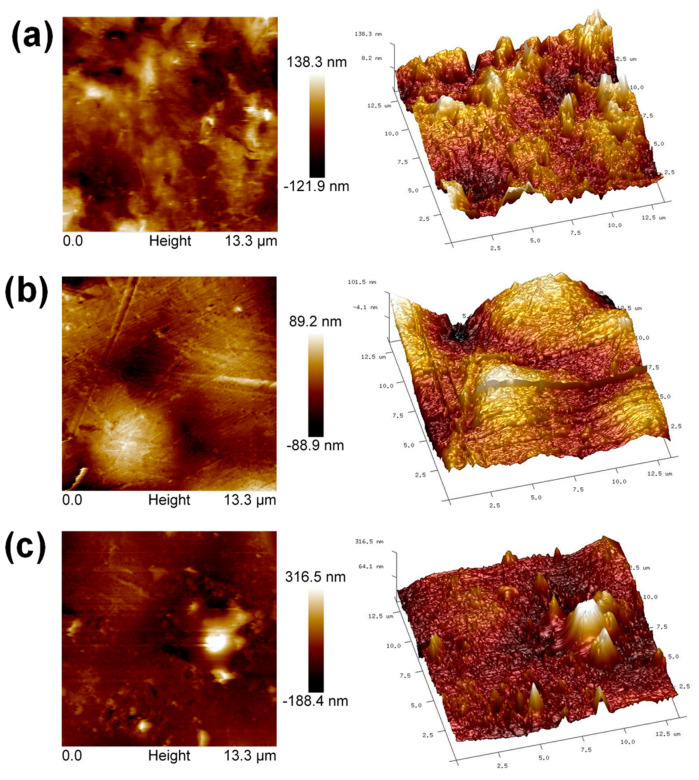
AFM height mapping 2D and 3D images: (**a**) PP film, (**b**) PP@NiO hybrid film, and (**c**) PP@NiFe_2_O_4_ hybrid film.

The surface roughness of the films was measured using atomic force microscopy (AFM). Figure 10 shows AFM height mapping 2D and 3D images, in which it is possible to observe differences in the topography of the hybrid film surfaces compared to the PP film. The mean square roughness (Rms) was obtained from the average of three different lines on the surface of each sample, obtaining Rms values of 26 ± 8 nm, 34 ± 3 nm, and 44 ± 1 nm for PP, PP@NiO and PP@NiFe_2_O_4_, respectively. These values suggest that the surface roughness tends to increase by incorporating NiO and NiFe_2_O_4_ NPs into the PP polymeric matrix. However, the homogeneity of the roughness is favored in the hybrid films, according to what is observed in the 3D image (Figure 10) and by the standard deviation values of the Rms measurements. Therefore, the roughness induced by the NPs into the PP matrix occurs mainly at the nanometric level, as it is expected for hybrid materials where their interactions occur at the molecular level or at the nanoscale.

### 2.5. Hydrophilicity of the Films

**Figure 11:**
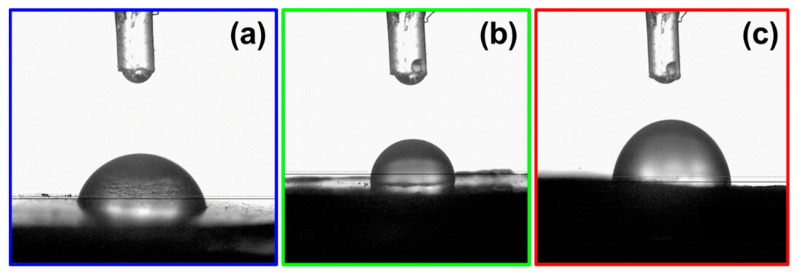
Optical photographs of the water droplet shape used for measuring the contact angle values: (**a**) PP film, (**b**) PP@NiO hybrid film, and (**c**) PP@NiFe_2_O_4_ hybrid film.

The contact angle technique was used to determine the hydrophilicity of the PP film, as well as the hybrid films PP@NiO and PP@NiFe_2_O_4_. Results showed that the contact angle average for PP, PP@NiO, and PP@NiFe_2_O_4_ films was 71° ± 1.0, 75° ± 1.0, and 81° ± 1.5, respectively. All the films were hydrophilic since their contact angle values fell between 10° < θ < 90°. On the other hand, Figure 11 shows the optical photographs of the water droplet shape. From these photographs, it is possible to observe differences between PP film and hybrid films because a more spherical droplet is observed on the surfaces of the hybrid films. This behavior indicates an increased contact angle due to the NPs’ hydrophobicity when introduced into the PP polymeric matrix. This result suggests the presence of the nanoparticles at the film interface. The behavior described above is related to the Rms roughness values obtained for the hybrid films studied in this research; a higher roughness value increases the contact angle value. This is in accordance with previous work [26], where L-B films of fatty acids on a glass substrate were evaluated, and where, by varying the micro-roughness of the solid surface, effects on the wetting dynamics were observed. This has been interpreted using the kinetic molecular theory of wetting and suggests that wetting is a decreasing function of microroughness; specifically, increasing Rms microroughness increases the activation free energy of wetting.

### 2.6. Antibacterial Assay of the Films

**Figure 12:**
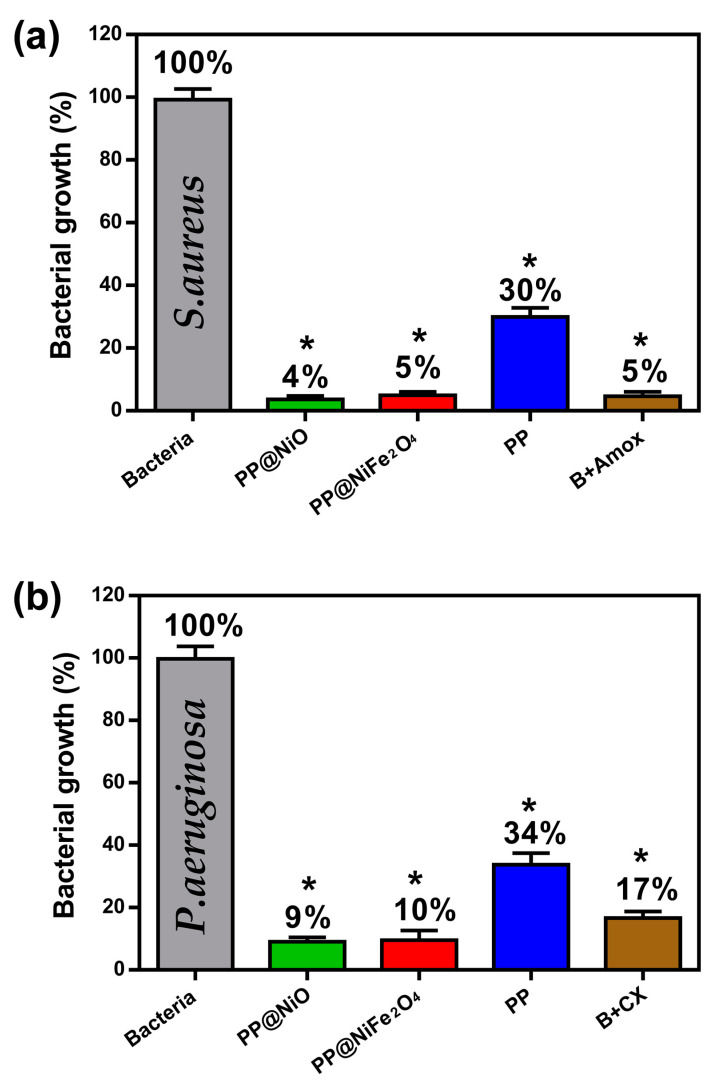
Bacterial growth (%) vs. PP film, PP@NiO, and PP@NiFe_2_O_4_ hybrid films graphs (**a**) for *S. aureus* and (**b**) for *P. aeruginosa*. * = significance shown at a 95% confidence level.

After characterizing the films, the antibacterial susceptibility against *S. aureus* and *P. aeruginosa* strains was studied. We used 10^2^ growth cells and analyzed the absorption intensity of the wells at λ = 595 nm, which is a standard value for bacteria. In this test, the absorption intensity is proportional to the bacterial growth. Figure 12 displays the bacterial growth graphs for *S. aureus* and *P. aeruginosa* in PP film, PP@NiO, and PP@NiFe_2_O_4_ hybrid films. The graphs include positive controls (bacteria + (amoxicillin for *S. aureus* and ciprofloxacin for *P. aeruginosa*)) and negative controls (bacteria without external agents). In addition, compared with the positive control, the significance per system is presented (represented by (*), with * = significance shown at a 95% confidence level, developed by the Tukey test with the GraphPad Prism 8 Software).

According to the graphs (Figure 12), inhibition of bacterial growth for *S. aureus* was 96%, 95%, and 70%, for PP@NiO, PP@NiFe_2_O_4_, and PP, respectively. In the case of *P. aeruginosa*, the inhibition was 91%, 90%, and 66% by PP@NiO, PP@NiFe_2_O_4_, and PP, respectively. In general, all films evaluated displayed significant antibacterial activity. However, the hybrid films exhibited a higher inhibitory effect than the PP film, likely due to the synergistic interaction between the NPs and the PP polymer. It has been observed that the antibacterial activity of the evaluated films can be on par with that of traditionally used antibiotics. Based on the findings and theories presented in the literature [9,27], it is suggested that the antibacterial properties of the hybrid films against bacteria involve multiple processes. These processes include the presence of radicals, the removal of essential metals from their original binding sites, and ligand interactions. A crucial observation is that metal ions play a vital role in the antibacterial effect, regardless of the specific mechanism. Therefore, if a material can release metal ions beyond a specific threshold, it will exhibit antibacterial activity. The greater the number of ions released, the stronger the antibacterial effect of the material.

### 2.7. Cytotoxicity of the Films

**Figure 13:**
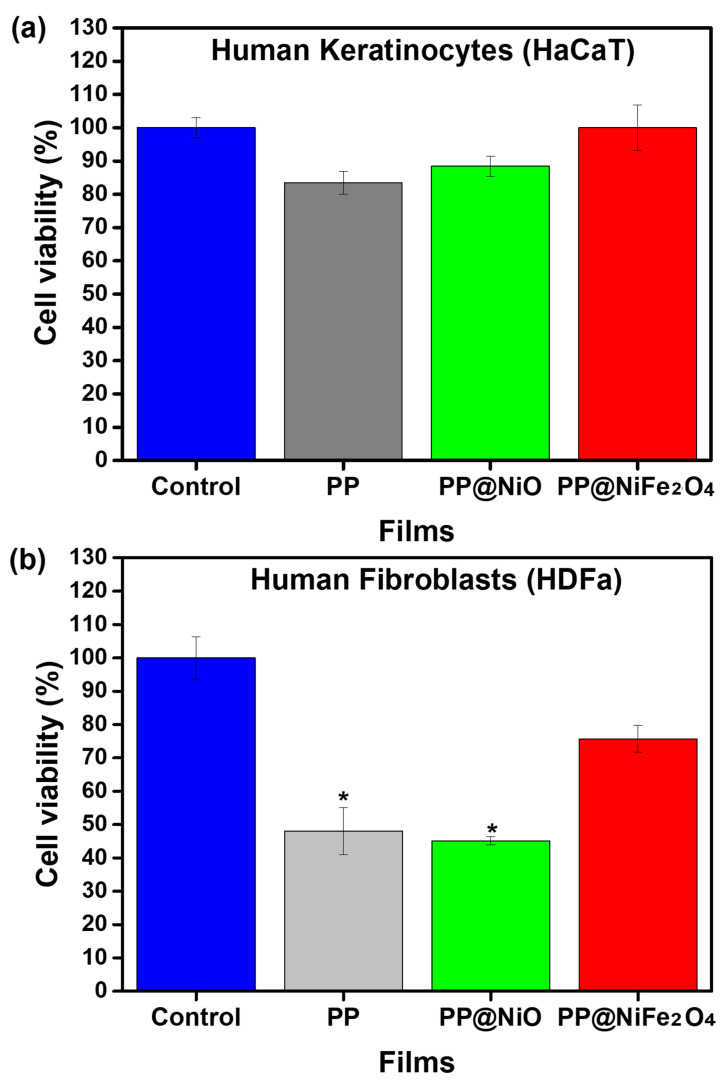
Graphs of cell viability vs. PP, PP@NiO, and PP@NiFe_2_O_4_ films for human (**a**) keratinocytes and (**b**) fibroblasts. *, *p* < control cells. Cells were evaluated in contact with the lixiviated products of the different films.

The potential cytotoxicity effect of the obtained films was assessed by the cytotoxicity index (IC50), a quantitative measurement indicating the amount of a certain material that is required in vitro for a biological component to decrease by 50%. For this trial, two different cells were evaluated, keratinocytes and fibroblasts, with keratinocytes being the main cellular component found in the epidermis (the outer layer of the skin) and fibroblasts in the dermis (the inner layer of the skin). Figure 13 shows the cytotoxicity results of the PP film, PP@NiO, and PP@NiFe_2_O_4_ hybrid films against the cells evaluated.

Based on the results obtained, the lixiviated products of PP films are biocompatible with human keratinocytes, with a cell viability percentage of 100%, 88%, and 83% for the PP@NiFe_2_O_4_, PP@NiO and PP films, respectively. This is particularly valuable since the hybrid materials obtained from this research can be used as antibacterial coating agents on various surfaces. It is noteworthy that keratinocytes are the most exposed cells of the skin and, thus, are the first point of contact with a barrier surface. Therefore, the biocompatibility of these materials with keratinocytes is highly desirable. The lixiviated products of PP and PP@NiO films decreased the cell viability of human fibroblasts to 45% and 48%, respectively. On the other hand, the PP@NiFe_2_O_4_ film was found to be biocompatible, exhibiting 75% cell viability for fibroblasts. This indicates that the addition of NiFe_2_O_4_ NPs to the PP polymeric matrix significantly improves its biocompatibility. To make use of the antibacterial properties of the PP@NiO hybrid film, it is crucial to establish a correlation between the concentration of the polymer and NPs so that there is significant antibacterial activity without compromising biocompatibility, particularly for fibroblasts. It is important to study the cytotoxicity of isolated NPs, as they may eventually be released from the polymeric matrix and encounter human tissues via the skin. Our research group has conducted previous studies on this topic [18], showing that NiO and NiFe_2_O_4_ NPs are biocompatible with human keratinocytes and fibroblasts. For future studies, we propose evaluating different concentrations of the percentage in weight of NPs/polymer, as well as to study the influence of the concentration of NPs in the polymeric matrix on its antibacterial activity and cytotoxicity.

## 3. Discussion

Through a simple, affordable, and reproducible methodology, this research produced PP, PP@NiO, and PP@NiFe_2_O_4_ films. Various spectroscopic and microscopic characterization techniques were used to confirm the successful incorporation of nanoparticles into the polymeric matrix, resulting in hybridized PP@NiO and PP@NiFe_2_O_4_ films. X-ray diffraction analysis revealed the characteristic peaks of PP in all the films obtained. The hybrid films exhibited the characteristic diffraction peaks of NiO and NiFe_2_O_4_ based on their corresponding crystallographic cards, indicating that the nanoparticles were indeed included in the PP polymeric matrix. The average crystallite size for the hybrid films was 7 ± 0.4 nm and 6 ± 1.1 nm for PP@NiO and PP@NiFe_2_O_4,_ respectively. It can be confirmed that the nanocrystallite size of the previously synthesized nanoparticles has been preserved when incorporated into the PP polymeric matrix.

Using Raman spectroscopy, it was observed that the interaction between NiO and NiFe_2_O_4_ NPs with PP in hybrid films occurs primarily through CH_3_, CH, and the C-C carbon chain groups of the PP. The formation of a hybrid material occurred instead of a composite material. Meanwhile, when examining the hybrid films using scanning electron microscopy, we found that the PP polymer undergoes degradation at 30 kV. However, this degradation was not observed in the hybrid films at the same voltage. Therefore, the NiO and NiFe_2_O_4_ NPs provided chemical stability to the PP matrix. Furthermore, the chemical mapping demonstrated identifying and quantifying individual elements in each hybrid film. The nanoparticles were present in a *w*/*w* ratio of approximately 10% with respect to the polymer. It is worth noting that the obtained films were hydrophilic, which could potentially encourage bacteria interactions. However, introducing hybrid films increased the contact angle value, which is attributed to the increase in surface roughness. This could promote the adhesion of bacteria and encourage interaction with the NPs in the hybrid films, thereby facilitating their antibacterial action. The antibacterial efficacy of the films was tested against two bacterial strains: *S. aureus*, a Gram-positive bacterium, and *P. aeruginosa*, a Gram-negative bacterium. Hybrid films showed significant inhibition of bacterial growth, over 95% for *S. aureus* and over 90% for *P. aeruginosa*, for PP@NiO and PP@NiFe_2_O_4_, respectively. The inhibition percentages are at the level of the antibiotics traditionally used, especially for *P. aeruginosa*, a bacterium resistant to many antibiotics and difficult to combat. A synergistic effect was observed between NPs and PP, generating improved antibacterial activity. This behavior allows hybrid materials to be used as protective coatings on various surfaces, preventing the spread of bacterial infections. The PP films are biocompatible against keratinocytes and fibroblasts through cytotoxicity studies. This is especially important since keratinocytes are the most exposed cells of the skin. The PP@NiFe_2_O_4_ hybrid film exhibited enhanced biocompatibility with fibroblasts, making it an excellent candidate for surface applications due to its antibacterial properties and biocompatibility. Future research could focus on evaluating variations in the concentrations of NPs within the PP polymeric matrix to accomplish an optimal balance between antibacterial properties and biocompatibility.

## 4. Materials and Methods

### 4.1. Materials

Chemical reagents used for synthesis of the nanoparticles, that is, nickel acetate tetrahydrate (Ni(C_2_H_3_O_2_)_2_·4H_2_O) (98%), nickel acetylacetonate (Ni(C_5_H_7_O_2_)_2_) (95%), iron acetylacetonate (Fe(C_5_H_7_O_2_)_3_) (99.9%), sodium hydroxide (NaOH) (97%), and acetone (CO(CH_3_)_2_) (99.5%) were acquired from Sigma-Aldrich (St. Louis, MO, USA). A Barnstead E-pure deionization system was used to obtain deionized water (18 MΩ-cm^−1^).

For hybrid films synthesis, polypropylene (PP) and xylene (C_6_H_4_(CH_3_)_2_) (99%) were also acquired from Sigma-Aldrich.

Assays to test the antibacterial activity of the films were performed using Trypticase soy agar (TSA; BD Bioxon™ (Franklin Lakes, NJ, USA)) and Trypticase soy broth (TSB; BD Bioxon™).

Cytotoxic studies were performed by using Dulbecco’s Modified Medium Eagle F12 (DMEM-F12), fetal bovine serum (SFB), penicillin/streptomycin (antibiotic/antimycotic) 0.25%, trypsin-EDTA 0.25%, phosphate-buffered saline (PBS; Ph = 7.4), and 3-(4,5-dimethylthiazol-2-yl)-2,5-diphenyltetrazolium bromide (MTT-Formazan), all purchased from Gibco (Thermo Fisher, Waltham, MA, USA).

### 4.2. Nanoparticles Synthesis

NiO and NiFe_2_O_4_ NPs were synthesized following the process previously reported by Rincón–Granados et al. [12]. For NiO NPs, 124.4 mg (5 × 10^−4^ mol) of nickel acetate tetrahydrate (Ni(C_2_H_3_O_2_)_2_•4H_2_O) and 40.0 mg (1 × 10^−3^ mol) of sodium hydroxide (NaOH) were milled for 20 min. The resulting powder was washed with distilled water and acetone, dried at room temperature (RT), and heated to 400 °C for 2 h, transforming into a greenish-grey powder. An amount of 70.6 mg (1 × 10^−4^ mol) of nickel acetylacetonate (Ni(C_5_H_7_O_2_)_2_), 70.6 mg (2 × 10^−4^ mol) of iron acetylacetonate (Fe(C_5_H_7_O_2_)_3_), and 32.0 mg (8 × 10^−4^ mol) of NaOH were milled until an orange powder was obtained, which was heated at 400 °C for 2 h, turning it into a dark-red powder. Finally, this powder was washed with distilled water and acetone and let to dry at RT.

### 4.3. Hybrid Films Synthesis

**Figure 14:**
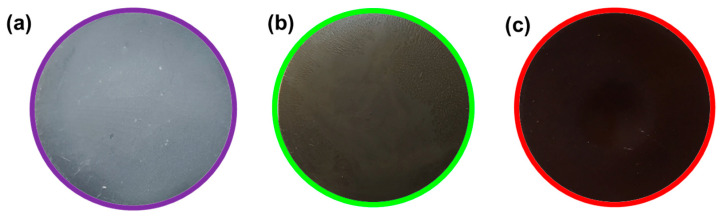
Photographs of (**a**) PP film, (**b**) PP@NiO hybrid film, and (**c**) PP@NiFe_2_O_4_ hybrid film.

A simple solvent casting method was utilized to obtain the hybrid films studied in the present work. Firstly, PP pellets were solubilized in p-xylene at 130 °C with constant stirring, heating the mixture close to its boiling point. Once dissolved, 10% *w*/*w* of the previously synthesized NPs were added, and the PP dissolution and NPs mixture was heated while stirring until the volume of the mixture was reduced to 20% of the original volume. The remaining was solvent evaporated at RT until a solid film was obtained. The macroscopic aspect of PP film and PP@NiO and PP@NiFe_2_O_4_ hybrid films can be observed in Figure 14.

### 4.4. Hybrid Films Characterization

X-ray diffraction patterns of the films were acquired by using an Empyrean PIXel 1D Malvern Panalytical diffractometer (Grovewood Road, UK), equipped with Cu Kα radiation (λ = 1.5406 Å); diffraction patterns between 10° and 70° were obtained using 2θ step of 0.01°, and acquiring time of 0.25 s per point. By means of the Debye-Scherer equation, D = Kλ /βcosθ, where K corresponds to the shape factor equal to 0.9, λ to the Cu Kα radiation, β to the full width at half maximum (FWHM) intensity of the selected peaks, and θ to the Bragg angle, the average crystal size, D, of the NPs was estimated from their corresponding diffractograms. Chemical characterization of the films was performed by acquiring the Raman spectra of the films and hybrid films from 200 to 3500 cm^−1^, using a WiTec Alpha 300 XR dispersive Raman spectrometer (Ulm, Germany) with 180° backscatter configuration for detection. The Excitation beam was an Nd: YVO_4_ 532 nm laser and incident power on the sample was 1 and 3 mW. Scanning electron microscopy (SEM) micrographs were obtained using an ESEM XL 30 Philips (Thermo Fisher, Waltham, MA, USA) equipment operating at 30 kV. High-resolution transmission electron micrographs (HR-TEM) were obtained with an FEI Tecnai F20 microscope (Thermo Fisher, Waltham, MA, USA), operating at 200 KV S/TEM field emission with an X-TWIN lens and a high brightness field emission electron gun (FEG). The films’ chemical mapping and elemental composition were attained using a JSM-7800F, JEOL (Akishima, Tokio, Japon), SEM equipment operated at 15 kV. For Atomic force microscopy, a microscope VEECO NANOSCOPE IV+MULTIMODE MMAFM/ST (NY, USA) was used. The tapping mode was employed with a scanning rate of 1 Hz and a scan resolution of 256 lines. The scan area was set as 13 μm × 13 μm. The tip was Tap300GD-G 300 kHz, 40 N/m. All the images obtained were processed with NanoScope Analysis v1.40 software, from which the topographic profiles were obtained, as well as roughness parameters. Water drops of 30 µL were used to determine contact angle values, and three different areas of the film’s surface were measured using a Pocket Goniometer Model PG-1 (Boston, MA, USA).

### 4.5. Antibacterial Assays

The antibacterial effect of hybrid films was evaluated using the aerobic bacteria obtained as lyophilized cultures from ATCC^®^ (Rockville, MD, USA): *P. aeruginosa* (43636™) and *S. aureus* (23235™). Both bacterial strains were individually cultured on Petri plates with TSA (Trypticase Soy Agar 20 g, 500 mL H_2_O) and incubated at 35 °C under aerobic conditions for 24 h. The growth of pure cultures of each strain was collected from agar surfaces and resuspended in TSB (Trypticase Soy Broth). To obtain a bacterial suspension with a cell density of 10^9^ cells/mL, each suspension was adjusted to an optical density (OD) of 1 at λ = 600 nm, and then, serial dilutions were performed to obtain bacterial suspensions of 10^2^ cells/mL. The susceptibility of *P. aeruginosa* and *S. aureus* after contact with the polymer@NPs films was studied using the broth dilution method following the ISO 22196 standard [27] and using TSB as broth.

Sterilized autoclaved samples (121 °C for 20 min) with a surface area of 0.8 cm^2^ were cut into four equal parts and individually placed in 12-well culture plates, and each sample was inoculated with 600 μL of bacterial suspension (10^2^ cells/mL) and incubated at 35 °C in an orbital shaker incubator at 60 rpm. Negative control (bacterial suspension with no film) and positive control (300 µL of bacterial suspension + 300 µL of amoxicillin for *S. aureus* or 300 µL of ciprofloxacin for *P. aeruginosa*), per assay, were added. After 24 h of incubation, the wells were rinsed three times with fresh TSB. Finally, 600 μL of TSB was added, homogenizing by micropipette, and 200 μL of this TSB supernatant was transferred in a 96-well culture plate to read the OD at λ = 595 nm. The measured OD of supernatants is directly proportional to the number of bacteria present in the broth. All evaluated materials were tested in triplicate.

### 4.6. Instruments for Antibacterial Assays

The optical density measurements performed during the antibacterial assays were determined using an Ultraviolet-Visible (UV-Vis) spectrophotometer FilterMax F5 Multi-mode microplate reader from Molecular Devices. The bacterial cultures were incubated in an orbital shaker incubator Cleaver Scientific Ltd. (Rugby, UK).

### 4.7. Cytotoxic Assays

The cytotoxic effect of the released products from the hybrid films was assessed by the MTT-Formazan assay using human dermal fibroblast (HDFa; ATCC^®^, PCS-201-012™) and human epidermal keratinocytes (HaCaT; CLS Cell Lines Service, 300493). Confluent cultures of HDFa and HaCaT were independently treated with 0.05% trypsin-EDTA, collected by centrifugation, and suspended in DMEM-F12 complemented with 10% v/v of SFB, 1% *v*/*v* of antibiotic/antimycotic (DMEM-F12/c). Each cell type was individually seeded on 48-well culture plates at 5 × 10^3^ cells/well with DMEM-F12/c, and incubated for 24 h at 37 °C, 95% humidity, and 5% CO_2_. To obtain the released products of the PP, PP@NiO, and PP@NiFe_2_O_4_ films, the films were sterilized by UVC radiation and then were immersed in DMEM-F12/c in a *w*/*v*. ratio of 21 mg/mL and incubated for 24 h.

After the incubation period, supernatants containing the lixiviated products of the films were individually collected and transferred to the cell-seeded wells, replacing the initially used media, and the 48-well culture plates were returned to the incubator. Cell-seeded wells with fresh cultured media were used as positive controls. After 24 h of incubation, the cell cultures were gently washed twice with PBS and incubated with a solution of DMEM-F12/c with MTT (1:10) at 37 °C under darkness. After 3 h of incubation, the DMEM-F12/c:MTT solution was discarded, and the formazan crystals produced by the metabolically active cells were dissolved in ISO:DMSO solution (1:1). The *OD* at λ = 570 nm of the formazan dissolutions was measured, and the cell viability in percentage was calculated using the following equation:

$$Cell\;viability\;\left(\%\right)=\left[\frac{\left(OD_{A1}-OD_{B1}\right)}{\left(OD_{A2}-OD_{B1}\right)}\right]\times 100$$

where *OD_A_*_1_ is the absorbance of the formazan dissolution produced by cells incubated with the lixiviated products from the different films, *OD_A_*_2_ is the absorbance of the formazan dissolution produced by the cells incubated only with fresh culture media (positive control), and *OD_B_*_1_ is the absorbance of ISO:DMSO blank solution (1:1).

### 4.8. Instruments for Cytotoxic Assays

The MTT assay studied the viability of cells exposed to the lixiviated products from the films. The optical density of formazan dissolutions was measured in a spectrophotometer multi-mode microplate reader synergy™ HTX BioTek^®^.

### 4.9. Statistical Analysis

The data obtained from biological experiments were analyzed using the GraphPad Prism Software. The statistical significance was determined by the one-way analysis of variance (ANOVA) and Tukey’s post hoc test, considering *p* < 0.05 as statistically significant.

## 5. Conclusions

A simple and cost-effective process was employed to obtain PP@NiO and PP@NiFe_2_O_4_ hybrid films. Using spectroscopic and microscopic techniques, we have confirmed the composition and structure of the hybrid films and the successful integration of nanoparticles into the polymeric matrix. The presence of NPs in the films increased the contact angle value due to the rise in surface roughness. This could facilitate their interaction with different bacterial strains as the films are hydrophilic. Our tests have shown that these films promote effective antibacterial action against two main resistant bacteria, *S. aureus* and *P. aeruginosa,* as evaluated in vitro. Additionally, we have corroborated that these hybrid films are biocompatible with keratinocytes, and the PP@NiFe_2_O_4_ film is also biocompatible with fibroblasts. These hybrid films can potentially be highly effective antibacterial surface materials, inhibiting bacterial growth and early stages of biofilm formation and preventing bacterial spread.

## Data Availability

Data are contained within the article and supplementary materials.

## Supplementary Materials

The supporting information can be downloaded at: https://www.mdpi.com/article/10.3390/ijms242317052/s1.

## References

[1] Varela M.F., Stephen J., Lekshmi M., Ojha M., Wenzel N., Sanford L.M., Hernandez A.J., Parvathi A., Kumar S.H. (2021). Bacterial Resistance to Antimicrobial Agents. Antibiotics.

[2] Deurenberg R.H., Stobberingh E.E. (2008). The Evolution of Staphylococcus Aureus. Infect. Genet. Evol..

[3] Zakaria Z., Sreenivasan S., Mohamad M. (2019). Antimicrobial Activity of Piper Ribesoides Root Extract Against Staphylococcus Aureus. J. Appl. Biol. Sci..

[4] Papadimitriou-Olivgeris M., Jacot D., Guery B., Filloux A., Ramos J.-L. (2022). How to Manage Pseudomonas Aeruginosa Infections. Pseudomonas Aeruginosa: Biology, Pathogenesis and Control Strategies.

[5] Labovská S., Das T. (2021). Pseudomonas Aeruginosa as a Cause of Nosocomial Infections. Pseudomonas Aeruginosa.

[6] Driscoll J.A., Brody S.L., Kollef M.H. (2007). The Epidemiology, Pathogenesis, and Treatment of Pseudomonas Aeruginosa Infections. Drugs.

[7] Azam A., Ahmed A.S., Oves M., Khan M.S., Memic A. (2012). Size-Dependent Antimicrobial Properties of CuO Nanoparticles against Gram-Positive and -Negative Bacterial Strains. Int. J. Nanomed..

[8] Hajipour M.J., Fromm K.M., Akbar Ashkarran A., Jimenez de Aberasturi D., de Larramendi I.R., Rojo T., Serpooshan V., Parak W.J., Mahmoudi M. (2012). Antibacterial Properties of Nanoparticles. Trends Biotechnol..

[9] Vázquez Olmos A., Vega Jiménez A., Paz Díaz B. (2018). Mecanosíntesis y Efecto Antimicrobiano de Óxidos Metálicos Nanoestructurados. Mundo Nano.

[10] Ilbeigi G., Kariminik A., Moshafi M.H. (2019). The Antibacterial Activities of NiO Nanoparticles Against Some Gram-Positive and Gram-Negative Bacterial Strains. Int. J. Basic. Sci. Med..

[11] Rincón-Granados K., Vázquez-Olmos A., Rodriguez-Hernandez A., Vega-Jiménez A., Ruiz-Ruiz V.-F., Garibay-Febles V., Ximenez-Fyvie L.A. (2021). Facile Solid-State Synthesis and Study in Vitro of the Antibacterial Activity of NiO and NiFe_2_O_4_ Nanoparticles. Materialia.

[12] Bhosale S.V., Ekambe P.S., Bhoraskar S.V., Mathe V.L. (2018). Effect of Surface Properties of NiFe_2_O_4_ Nanoparticles Synthesized by Dc Thermal Plasma Route on Antimicrobial Activity. Appl. Surf. Sci..

[13] Stankic S., Suman S., Haque F., Vidic J. (2016). Pure and Multi Metal Oxide Nanoparticles: Synthesis, Antibacterial and Cytotoxic Properties. J. Nanobiotechnology.

[14] Oskam G. (2006). Metal Oxide Nanoparticles: Synthesis, Characterization and Application. J. Solgel Sci. Technol..

[15] Taheri-Ledari R., Maleki A. (2022). 1—Classification of Micro and Nanoscale Composites. Heterogeneous Micro and Nanoscale Composites for the Catalysis of Organic Reactions.

[16] Oprea M., Panaitescu D.M. (2020). Nanocellulose Hybrids with Metal Oxides Nanoparticles for Biomedical Applications. Molecules.

[17] Sathya S., Murthy P.S., Devi V.G., Das A., Anandkumar B., Sathyaseelan V.S., Doble M., Venugopalan V.P. (2019). Antibacterial and Cytotoxic Assessment of Poly (Methyl Methacrylate) Based Hybrid Nanocomposites. Mater. Sci. Eng. C.

[18] Rincon-Granados K.L., Vázquez-Olmos A.R., Rodríguez-Hernández A.-P., Prado-Prone G., Garibay-Febles V., Almanza-Arjona Y.C., Sato-Berrú R.Y., Mata-Zamora E., Silva-Bermúdez P.S., Vega-Jiménez A. (2023). Bactericidal and Cytotoxic Study of Hybrid Films Based on NiO and NiFe_2_O_4_ Nanoparticles in Poly-3-Hydroxybutyrate. J. Clust. Sci..

[19] Sastri V.R. (2010). Plastics in Medical Devices.

[20] Santhoshkumar J., Kumar S.V., Rajeshkumar S. (2017). Synthesis of Zinc Oxide Nanoparticles Using Plant Leaf Extract against Urinary Tract Infection Pathogen. Resour. Effic. Technol..

[21] Paleo A.J., Krause B., Mendes A.R., Tavares C.J., Cerqueira M.F., Muñoz E., Pötschke P. (2023). Comparative Thermoelectric Properties of Polypropylene Composites Melt-Processed Using Pyrograf^®^ III Carbon Nanofibers. J. Compos. Sci..

[22] Nielsen A.S., Batchelder D.N., Pyrz R. (2002). Estimation of Crystallinity of Isotactic Polypropylene Using Raman Spectroscopy. Polymer.

[23] Arruebarrena de Báez M., Hendra P.J., Judkins M. (1995). The Raman Spectra of Oriented Isotactic Polypropylene. Spectrochim. Acta A Mol. Biomol. Spectrosc..

[24] Bose P., Ghosh S., Basak S., Naskar M.K. (2016). A Facile Synthesis of Mesoporous NiO Nanosheets and Their Application in CO Oxidation. J. Asian Ceram. Soc..

[25] Ahmed A.A., Hashim M.R., Rashid M. (2019). Control of the Structural, Electrical and Optical Properties of Spin Coated NiO Films by Varying Precursor Molarity. Thin Solid Film.

[26] Semal S., Blake T.D., Geskin V., de Ruijter M.J., Castelein G., De Coninck J. (1999). Influence of Surface Roughness on Wetting Dynamics. Langmuir.

[27] Palza H., Quijada R., Delgado K. (2015). Antimicrobial Polymer Composites with Copper Micro- and Nanoparticles: Effect of Particle Size and Polymer Matrix. J. Bioact. Compat. Polym..

