# Supplementary material for: Antibacterial and Cytotoxic Study of Hybrid Films Based on Polypropylene and NiO or NiFe2O4 Nanoparticles
Source: Int J Mol Sci. 2023 Dec 2;24(23):17052. doi: 10.3390/ijms242317052 (PMC10707088; doi:10.3390/ijms242317052)
Supplement: Supplementary file 1 [file ijms-24-17052-s001.zip › ijms-2671963-supplementary.pdf]

Supporting Information

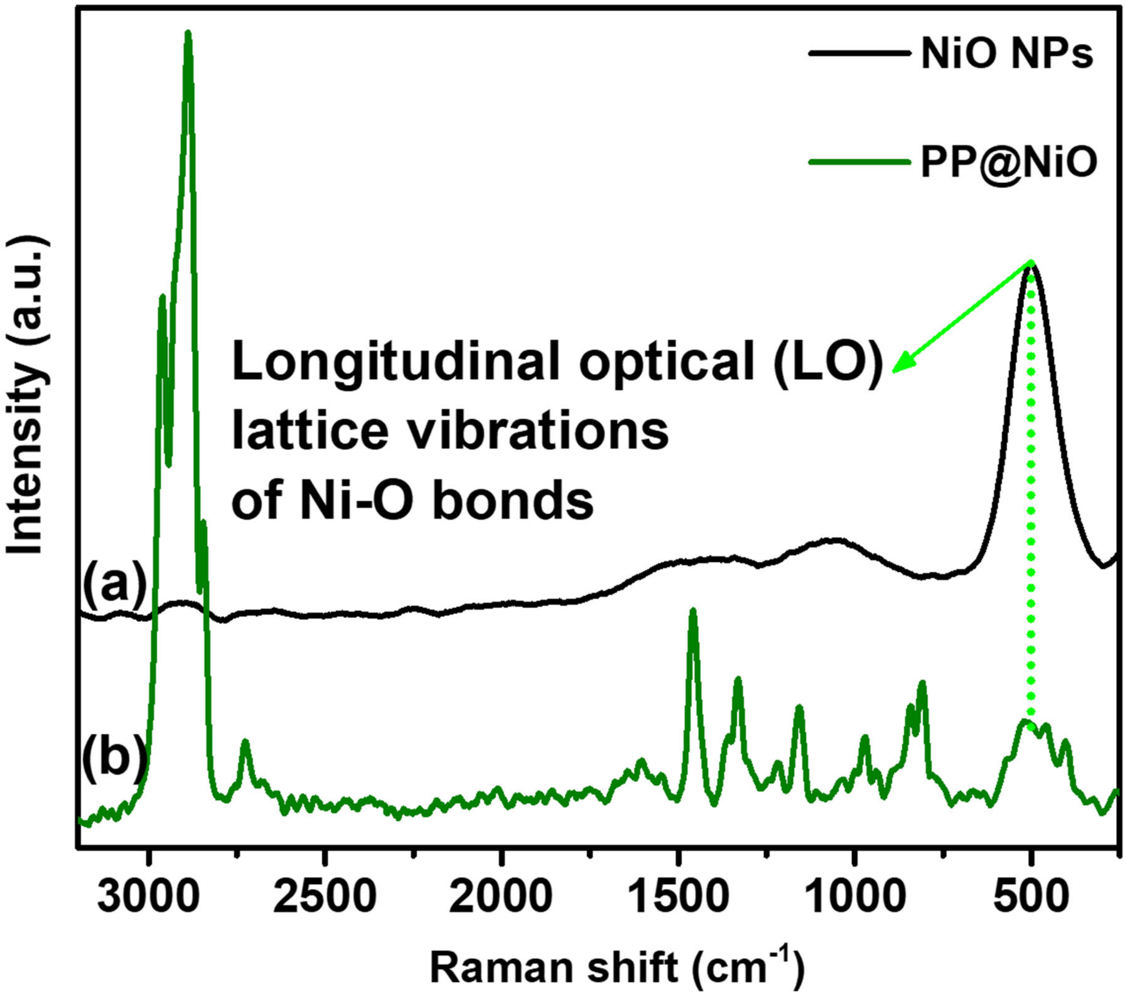

Figure S1. Raman spectra of (a) NiO nanoparticles and (b) PP@NiO hybrid film.

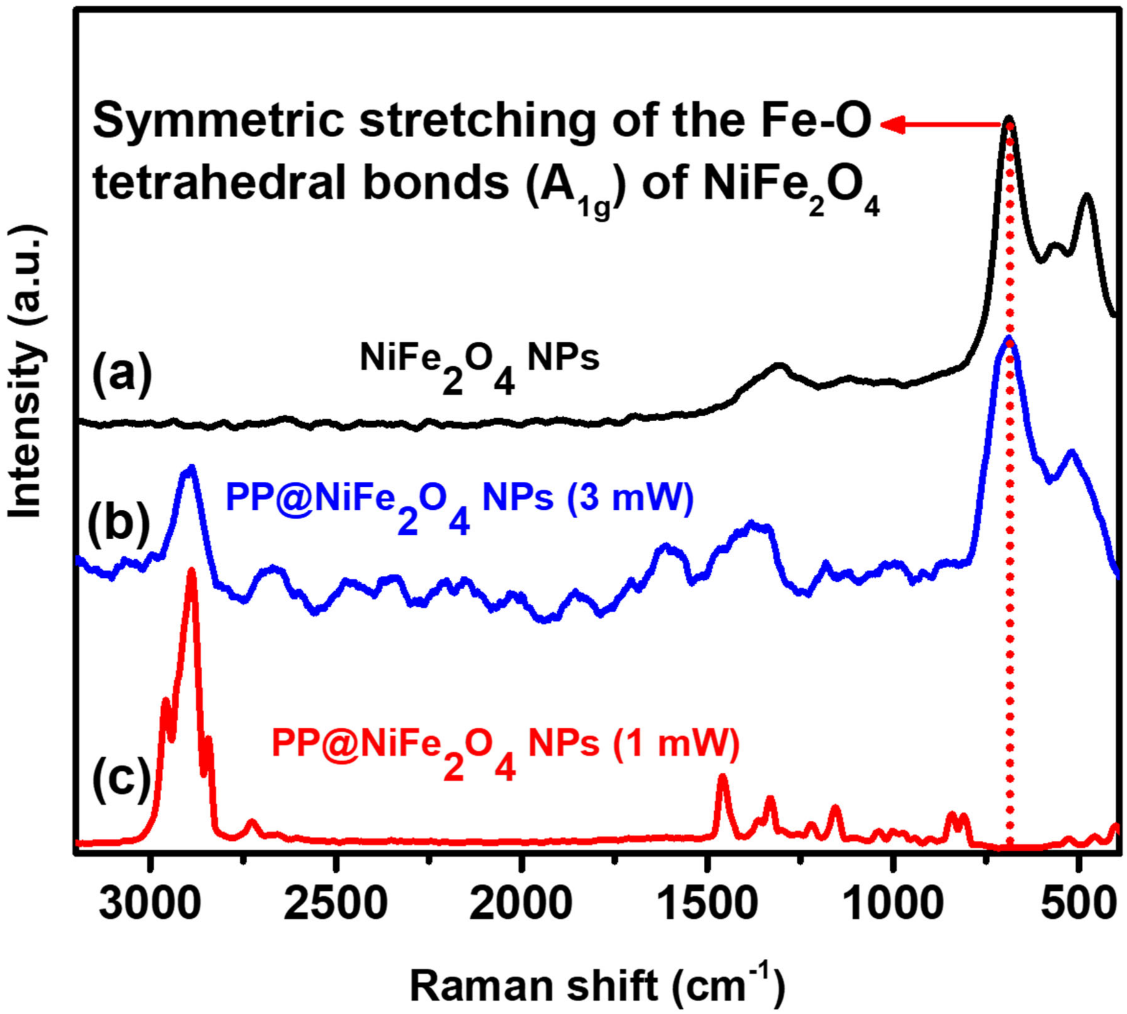

Figure S2. Raman spectra of (a) $NiFe_2O_4$ nanoparticles (at 3 mW laser power), (b) PP@$NiFe_2O_4$ hybrid film (at 1 mW laser power), and (c) PP@$NiFe_2O_4$ hybrid film (at 3 mW laser power).
